# Religiosity and Self-Rated Health as Predictors of Well-Being and Depression in Older Adults

**DOI:** 10.1093/geroni/igaf122.3129

**Published:** 2025-12-31

**Authors:** Nicholas Prefontaine, Andrew Revell

**Affiliations:** University of Massachusetts Dartmouth, Dartmouth, Massachusetts, United States; University of Massachusetts Dartmouth, Dartmouth, Massachusetts, United States

## Abstract

Depression and the well-being of older adults is crucial to public health, as this population faces heightened vulnerability to mental health challenges (Wang et al., 2022). In this present study, the role of religion and self-rated health (SRH) concerning depression and subjective well-being (SWB) were examined. Specifically, the objective of this examination was to identify the predictive nature of religiosity and SRH in a cohort of older adults aged 65 and older (M = 74) as a secondary data analysis. Data were drawn from the Religion, Aging, and Health Survey (RAHS; Krause, 2018), which included 1,500 participants, all Christian (N = 1,500), predominantly White (n = 748) and African American (n = 752), sampled through stratified sampling methods. Measures included Private Religious Practices, Self-Rated Health, and the Center for Epidemiological Studies Depression Inventory (CESD). Life satisfaction, self-esteem, and hope/optimism scales were used to assess SWB. Linear regression analyses indicated religiosity positively predicted SWB variables, such as life satisfaction, t(1220) = 8.81, p = .001, self-esteem, t(1313) = 6.07, p = .001, and hope/ optimism, t(1167) = 11.09, p = .001. Results further indicated that religiosity may offer meaning and purpose, enhancing SWB. Relationships with SRH were also explored. Future research would ideally be longitudinally designed to grasp how religiosity could impact individuals over time and include diverse populations to better understand how religiosity and SRH affect older adults’ health.

